# The (Poly)phenolic Profile of Separate Winery By-Products Reveals Potential Antioxidant Synergies

**DOI:** 10.3390/molecules28052081

**Published:** 2023-02-22

**Authors:** Antonio Costa-Pérez, Sonia Medina, Paola Sánchez-Bravo, Raúl Domínguez-Perles, Cristina García-Viguera

**Affiliations:** 1 Laboratorio de Fitoquímica y Alimentos Saludables (LabFAS), Departmento de Ciencia y Tecnología de Alimentos, CEBAS-CSIC, Campus of the University of Murcia-25, 30100 Espinardo, Murcia, Spain; 2 Department of Food Technology, EPSO, University Miguel Hernández, Ctra Beniel km 3.2, 03312 Orihuela, Alicante, Spain

**Keywords:** winery wastes, grape stems, grape pomace, wine lees, phenolic compounds, metabolic diversification, complementarity

## Abstract

The by-products of grapes (*Vitis vinifera* L.) in the winemaking process present a diverse phytochemical profile of (poly)phenols, essentially represented by phenolic acids, flavonoids, and stilbenes, which have health benefits. In winemaking, solid (grape stems and pomace) and semisolid (wine lees) by-products are generated, negatively impacting the sustainability of the agro-food activity and the local environment. Although information on the phytochemical profile of grape stems and pomace has been reported, especially information concerning (poly)phenols, research on wine lees is necessary to take advantage of the compositional traits of this residue. So, in the present work, an updated, in-depth comparison of the (poly)phenolic profiles of these three resulting matrices in the agro-food industry has been carried out to provide new knowledge and interesting data on the action of yeast and lactic acid bacteria (LAB) metabolism in the diversification of phenolic composition; additionally, we extract complementarities for the possible joint application of the three residues. The phytochemical analysis of the extracts was carried out using HPLC-PDA-ESI-MSn. The (poly)phenolic profiles of the residues showed significant discrepancies. The results obtained showed that the greatest diversity of (poly)phenols was found in the stems of the grapes, followed closely by the lees. Through technological insights, it has been suggested that yeasts and LAB, responsible for the fermentation of must, might play a key role in the transformation of phenolic compounds. This would provide new molecules with specific bioavailability and bioactivity features, which might interact with different molecular targets and, consequently, improve the biological potential of these underexploited residues.

## 1. Introduction

Grape (*Vitis vinifera* L.) is one of the most extensive crops worldwide, producing more than 78.0 million tonnes (Mt) globally in 2020. This crop is of special relevance in the countries of the Mediterranean Basin, especially Italy, Spain, France, and Turkey (8.2, 6.8, 8.9, and 4.2 Mt in 2020, respectively) (FAO, https://www.fao.org/faostat, accessed on 15 October 2022), where traditional wine consumption is strongly tied to regional and cultural factors [1]. Indeed, this agro-food industry is responsible for up to 46.6% of the total grape production, while up to 30.0% of the plant material used by this industry is discarded, constituting pollutant semisolid (wine lees) and solid (grape stems and pomace biomass) residues that negatively impact the local environment (mainly due to their high carbon content (31–54%) [2]). So, designing new valorization alternatives to recover and recycle winery by-products into new added-value co-products in light of the “circular economy” concept constitutes a critical research focus that can contribute to sustainability at a global level [1,3].

Valorization alternatives include soil correction, livestock feed, and ethanol or grape seed oil production [4,5]. Nonetheless, these applications do not allow for obtaining added value and only make a limited contribution to the competitiveness of the winery agro-food industry. Conversely, over the last few decades, the (poly)phenolic profiles of winery by-products (mainly grape stems and pomace) and more recently, wine lees [6,7], have been described, providing information on the occurrence of phenolic acids, catechins, flavonols, stilbenes, and anthocyanins [6]. The diversity and high concentration of these compounds in these winery by-products have suggested a broad range of functions related to plant physiology to overcome environmental challenges [8]. Their biological properties within the field of plant physiology prompted the study of their contribution to human health, such as benefits associated with their antioxidant, anti-mutagenic, anti-inflammatory, anti-carcinogenic, and antimicrobial activities [9,10,11,12,13,14,15,16,17]. Indeed, the occurrence of a wide diversity of bioactive phenolic compounds in these materials has sparked the interest of the scientific community and industrial actors, who are motivated by the possibility to add value to them to produce new functional ingredients in the workflow according to circular economy policies. However, even though these materials could have applications in new added-value products, which could benefit human health (sources of bioactive compounds), not all matrices have been properly characterized [18,19,20,21,22]. Therefore, a more exhaustive characterization of the (poly)phenolic composition of wine lees has been performed, as well as an exploration of its biology.

The present work aims at exploring diverse winery by-products (grape stems, pomace, and wine lees) and their different (poly)phenolic profiles, which may induce significant differences in terms of radical scavenging activity. To accomplish this, we analyze the (poly)phenolic profiles of grape stems and pomace, as well as wine lees, via HPLC-linear ion trap mass spectrometry (HPLC-PDA-ESI-MSn) and 2,2-diphenyl-1-picrylhydrazyl assays (DPPH). Moreover, their quantitative phenolic profiles are analyzed statistically by Pearson’s correlation and principal component analysis (PCA) to identify the compounds responsible for the antioxidant activity recorded and to identify possible synergies between the phenolic compounds present at different concentrations in the distinct residues, in some cases with the participation of the yeast and lactic acid bacteria (LAB) metabolism (grape pomace and wine lees).

## 2. Results and Discussion

**Figure 1:**
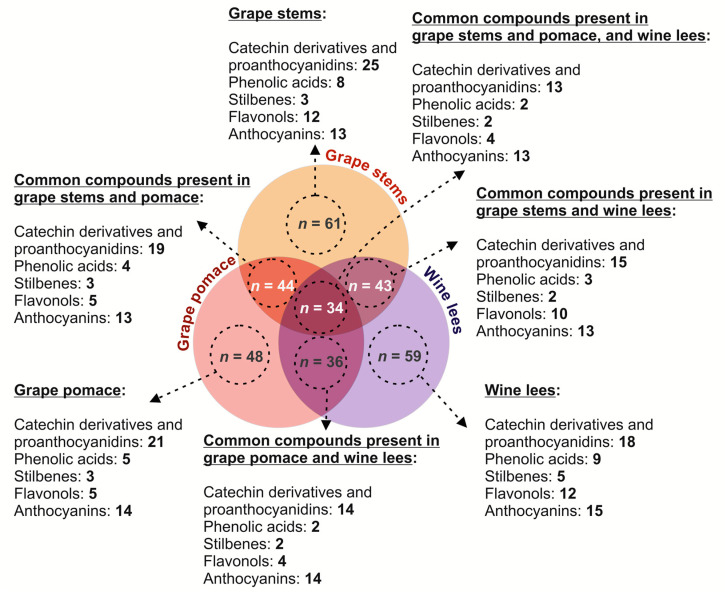
Schematic representation of the diversity of phenolic compounds identified in grape stems, grape pomace, and wine lees. Identity of the phenolic compounds.

The HPLC-PDA-ESI-MSn analysis of the hydromethanolic extracts obtained from grape stems, pomace, and wine lees revealed the occurrence of a broad range of (poly)phenols belonging to phenolic acids, catechin derivatives and proanthocyanidins, stilbenes, flavonols, and anthocyanins in the three matrices (Figure 1), showing a common and specific phenolic profile among the three matrices.

### 2.1. Comparative Analysis of the (Poly)phenolic Profile of Grape Stems, Grape Pomace, and Wine Lees

The analysis of the distribution of the different classes of phenolic compounds in grape stems and pomace, as well as wine lees (catechin derivatives and proanthocyanidins, phenolic acids, stilbenes, flavonols, and anthocyanins) suggest complementarity between all three materials, concerning their value as sources of bioactive phenolics. These differences could be responsible, to some extent, for the synergies that are explored in the present work, since they can help obtain new formulations, which would enhance their applicability as sources of health-promoting compounds. In this regard (Figure 1), the most abundant number of individual phenolics was found in grape stems and wine lees (*n* = 61 and *n* = 59, respectively), while only 48 phenolic compounds were identified in grape pomace. Catechin derivatives and proanthocyanidins, as well as anthocyanidins, were found to be the most ubiquitous compounds (Figure 1).

#### 2.1.1. Catechin Derivatives and Proanthocyanidins

**Figure 2:**
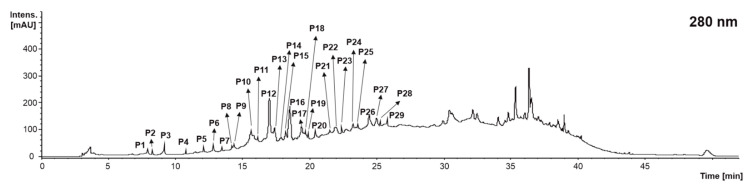
Chromatogram recorded at 280 nm representative of the catechin derivatives and proanthocyanidins (P) recorded in grape stems, grape pomace, and wine lees. Identification of the peaks annotated according to Table 1.

When analyzing the qualitative profile of the catechin derivatives and proanthocyanidins of grape stems, grape pomace, and wine lees corresponding to the vinification process for grape (*Vitis vinifera* L. var. ‘Monastrell’), 29 compounds were found in concentrations higher than the limit of detection (LOD) for the technique (Figure 2 and Table 1). According to this study, 25 catechin derivatives and proanthocyanidins were found in the grape stem, 21 in the grape pomace, and 19 in the wine lees.

When identifying the diverse catechin derivatives and proanthocyanidins present in grape stems, grape pomace, and wine lees, it was found that regardless of the isomers, 10 individual compounds were identified: proanthocyanidin trimer Type B (peaks **P1**, **P2**, **P13**, **P15**, **P17**, **P18**, **P25**, and **P29**), catechin-gallocatechin (peaks **P3**, **P4**, **P6**, and **P7**), gallocatechin (**P5**), proanthocyanidin dimer type B (peaks **P8**, **P9**, **P10**, **P11**, **P12**, **P14**, **P21**, and **P22**), proanthocyanidin dimer di-gallate (B type) (**P14**), catechin (**P16**), proanthocyanidin trimer monogallate (peaks **P19** and **P20**), epicatechin glucoside (**P26**), epicatechin (**P27**), and proanthocyanidin dimer monogallate (peaks **P23**, **P24**, and **P28**) (Table 1).

Peaks **P1**, **P2**, **P13**, **P15**, **P17**, **P18**, **P25**, and **P29** were isomeric compounds identified as proanthocyanidin trimer Type B (*n* = 8). All of them presented a [M − H]^−^ pseudo molecular ion at *m*/*z* 865 arbitrary mass units (amu), corresponding to the molecular formula C_45_H_38_O_18_. The fragment ion was at *m*/*z* 695 amu [M − H − 170]^−^ (loss of gallic acid) or *m*/*z* 577 amu [M − H − 288]^−^ (corresponding to the trimer upper subunit [23]), depending on the isomer considered, as well as the typical fragment at *m*/*z* 289 amu. This fragmentation pattern was consistent with the identification of the diverse isomers of B-type proanthocyanidin trimer in grape stems, grape pomace, and wine lees (Table 1). Nonetheless, only the grape stems presented all isomers; only **P1**, **P2**, **P13**, **P17**, **P18**, and **P25** were found the grape pomace, and **P2**, **P13**, **P18**, and **P24** in the wine lees. This reduction in the number of isomers of proanthocyanidin trimer (B type) in the grape pomace and wine lees could be due to the metabolic activity of the yeast burden characterizing the latter’s matrices [29]. Another trimer found in the winery by-products exhibited a [M − H]^−^ pseudo molecular ion at *m*/*z* 1017 amu, attributed to procyanidin trimer monogallate with the molecular formula C_52_H_42_O_22_ (peaks **P18** and **P20**) according to Rockenbach et al. (2012), based on the fragmentation pattern recorded at MS2 [M − H]^−^ which included ions at *m*/*z* 729, 677, 577, and 407 amu, linking the major fragments (*m*/*z* 729 and 577amu) to the loss of the (epi)catechin moiety [M − H − 288]^−^ and (epi)catechin gallate moieties [M − H − 441]^−^ [26]. In turn, the pseudo molecular ion at *m*/*z* 729 amu produced in MS3 [M − H]^−^ ions at 577 was linked to the loss of a galloyl group [M − H − 152] [24] (Table 1).

The LC-ESI-MSn analyses allowed us to identify four dimeric proanthocyanidins with [M − H]^−^ pseudo molecular ions at *m*/*z* 593 amu (molecular formula C_30_H_26_O_13_), *m*/*z* 577 amu (C_30_H_26_O_12_), *m*/*z* 881 amu (peak **P14**), and *m*/*z* 729 amu (peaks **P23**, **P24**, and **P28**). The compounds **P3**, **P4**, **P6**, and **P7** with pseudo molecular ions at *m*/*z* 593 amu displayed MS2 spectra, including product ions with *m*/*z* 441 amu [M − H − 152]^−^ (galloyl moiety) and 423 amu [M − H − 152 − 18]^−^ (galloyl moiety and water molecule) [23]. The compounds **P8**, **P9**, **P10**, **P11**, **P12**, **P14**, **P21**, and **P22** presented a [M − H]^−^ pseudo molecular ion at m/z 577 amu, indicating they are B-type proanthocyanidins formed by two (epi)catechins. The MS2 spectra showed the presence of product ions with *m*/*z* 425 [M − H − 152]^−^ (galloyl moiety) and 407 [M − H − 152 − 18]^−^ (galloyl moiety and water molecule) [23], as well as m/z 289 amu, indicating quinone methide or interflavan bond cleavage [25]. Additionally, [M − H]^−^ pseudo molecular ions at *m*/*z* 881 amu (peak **P14**) were detected and consistent with procyanidin dimer di-gallate (type B) (molecular formula C_44_H_34_O_20_) formed by two units of (epi)catechin and one unit of (epi)gallocatechin [23]. This tentative identification was completed by detecting MS2 fragments at *m*/*z* 577 amu, a dimeric proanthocyanidin indicating the C and D rings’ quinone methide fission; additionally, the fragment corresponding to the upper subunit was found to be the pseudo molecular ion *m*/*z* 303 amu. Additionally, although the fragment *m*/*z* 713 amu corresponding to the Diels–Alder fission in the C ring was not observed, the pseudo molecular ion generated was detected as a result of the loss of a water molecule (*m*/*z* 695 amu) [23]. Finally, [M − H]^−^ pseudo molecular ions at *m*/*z* 729 amu (peaks **P23**, **P24**, and **P28**) were recorded and tentatively identified as proanthocyanidin dimer monogallate via the MS2 spectra-producing ions at *m*/*z* 577 [M − H − 152]^−^ (galloyl moiety or a retro-Diels–Alder fission) and 559 [M − H − 152 + 18]^−^ (minus galloyl moiety, plus water molecule) [25] (Table 1).

Finally, the catechin derivatives gallocatechin (C_15_H_14_O_7_), catechin (C_15_H_14_O_6_), epicatechin glucoside (C2_1_H_24_O_11_), and epicatechin (C_15_H_14_O_6_) (peaks **P5**, **P16**, **P26**, and **P27**, respectively) were also identified on the basis of their [M − H]^−^ pseudo molecular ions, recorded at *m*/*z* 305, 289, 449, and 289, respectively. Gallocatechin generated fragments in MS3 at *m*/*z* 167 and 137 amu [24]. Moreover, the identification of these peaks was confirmed by matching them with the authentic standards.

For proanthocyanidin trimer type B, the distribution of the proanthocyanidin dimers identified in these compounds in the grape stems decreased in the grape pomace and wine lees in comparison with the former (potentially due to the metabolic capacity of native or added yeast and LAB), which break down complex molecules [29], except for proanthocyanidin dimer digallate, which was found only in the wine lees. This information indicates the value of grape stems, pomace, and wine lees as sources of such antioxidant compounds; although given the close relationship between their specific quantitative profiles and functional capacities, this should be confirmed by a correlation analysis.

#### 2.1.2. Phenolic Acids

**Figure 3:**
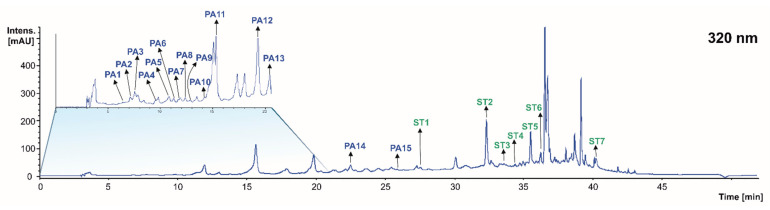
Chromatogram recorded at 320 nm representative of the phenolic acids (PA) and stilbenes (ST) recorded in grape stems, grape pomace, and wine lees. Identification of the peaks annotated according to Table 2 and Table 3, respectively.

When profiling the phenolic acids at 320 nm of the grape stems, pomace, and wine lees, 15 compounds were found in concentrations higher than the LOD for the technique (Figure 3 and Table 2). This tentative identification was carried out in the same way as in the previous section. Eight phenolic acids were found in the grape stem, five in the grape pomace, and nine in the wine lees.

Regardless of the isomers, 11 individual phenolic acids were identified, namely galloyl-hexoside (peaks **PA1** and **PA7**), gallic acid (peaks **PA2** and **PA3**), gentisic acid (**PA4**), protocatechuic acid-*O*-hexoside (peaks **PA5**, **PA6**, and **PA8**), caftaric acid (**PA9**), caftaric acid-glucuronide (**PA10**), caftaric acid derivative (**PA11**), *p*-coumaric acid pentoside (**PA12**), *p*-coumaric acid (**PA13**), ferulic acid pentoside (**PA14**), and gallic acid derivative (**PA15**) (Table 2).

According to the retention time, parent ion, and fragmentation pattern, the peaks **PA1** and **PA7** corresponded to the isomeric forms of galloyl-hexoside. Both of them presented a [M − H]^−^ pseudo molecular ion at *m*/*z* 331 amu, corresponding to the molecular formula C_13_H_16_O_10_. The major fragment obtained at *m*/*z* 169 amu [M − H − 162]^−^ (loss of hexose) is characteristic of the unesterified form of gallic acid [24,27]. Both isomers were found in the grape stems, while only isomer 1 was present in the grape pomace.

Peaks **PA2** and **PA3** were isomeric compounds identified as gallic acid that presented a [M − H]^−^ pseudo molecular ion at *m*/*z* 169 amu, corresponding to the molecular formula C_7_H_6_O_5_. The gallic acid isomers generated fragments in MS2 at *m*/*z* 125 amu [M − H − 44]^−^ (neutral loss of CO_2_), which is characteristic of this compound [28,30]. Both isomers were found in all three of the matrices analyzed (Table 2).

A [M − H]^−^ pseudo molecular ion at *m*/*z* 315 amu (peaks **PA5**, **PA6**, and **PA8)** was identified as protocatechuic acid-*O*-hexoside, with the molecular formula (C_13_H_16_O_9_). The MS2 spectra included the presence of ions at *m*/*z* 153 [M − H − 162]^−^ (loss of glucose) [33]. Isomers 1 and 3 were found only in the grape pomace and grape stems, respectively, while isomer 2 was present in both residues. No isomers of protocatechuic acid derivative were identified in the wine lees (Table 2).

Gentisic acid (C_7_H_6_O_4_), caftaric acid (C_13_H_12_O_9_), caftaric acid-glucuronide (C_15_H_16_O_10_), caftaric acid derivative, *p*-coumaric acid pentoside (C_14_H_17_O_7_), *p*-coumaric acid (C_9_H_8_O_3_), ferulic acid pentoside (C_15_H_19_O_8_), and ethyl gallate (peaks **PA4**, **PA9**, **PA10**, **PA11**, **PA12**, **PA13**, **PA14**, and **PA15**, respectively) were also identified on the basis of their [M − H]^−^ pseudo molecular ions, recorded at *m*/*z* 153, 311, 487, 623, 295, 162, 325 and 197, respectively, and molecular losses at MS2, according to previous descriptions in the literature [31,33]. Specifically, ethyl gallate (**PA15**) with the molecular formula (C_9_H_10_O_5_), generated an MS2 base peak at *m*/*z* 169 amu [M − H − 28]^−^ (neutral loss of CH_2_CH_2_) and MS3 fragment at *m*/*z* 125 amu (neutral loss of CO_2_) [31]. All these phenolic compounds are potentially ormed as a result of the metabolic activity of yeasts and LAB during winemaking [20]. Recently, ethyl gallate has been described in wine lees [7] as a particular product formed during the fermentation process by the esterification of gallic acid with ethanol [34], but it has also been detected in grape seeds, skin, and stems, such as gentisic acid (**PA4**) and *p*-coumaric acid (**PA13**) [31].

#### 2.1.3. Stilbenes

The assessment of the stilbene profile at 320 nm of the grape stems and pomace, as well as the wine lees revealed the occurrence of seven individual stilbenes in concentrations higher than the LOD for the HPLC-PDA-ESI-MSn-based analytical approach (Figure 3 and Table 3). The tentative identification of the stilbenes summarized in Table 3 was conducted according to the criteria specified in the Materials and Methods section.

Six individual stilbenes were identified, which were distributed in the separate matrices under study, namely oxyresveratrol-glucoside (**St1**), *trans* piceid isomer 1 (resveratrol hexoside) (**St2**), oxyresveratrol (**St3**), stilbenoid tetramer (Hopeaphenol) (**St4**), Σ-viniferin (isomers **St5** and **St7**), and *trans* piceid acid (**St6**) (Table 3).

The peaks **St1** and **St3** corresponding to oxyresveratrol and oxyresveratrol-glucoside (molecular formulas C_14_H_12_O_4_ and C_20_H_22_O_9_, respectively) exhibited [M − H]^−^ pseudo molecular ions at *m*/*z* 405 and 243 amu which allowed them to be detected in the wine lees, while they were absent in the grape stems and pomace (Table 3). The MS2 spectra of these compounds included deprotonated ions at *m*/*z* 243 amu [M − H − 162]^−^ (loss of a glucose molecule) and 224 [M − H − 19]^−^ amu (unspecific neutral loss, which was consistent with the stilbenes referred to in [27]). In addition, the presence of resveratrol glucoside, also known as *trans*piceid (**St2** and **St6**), corresponding to the molecular formula C_20_H_22_O_8_, was detected and featured a [M – H]^−^ pseudo molecular ion at *m*/*z* 389 amu that fragmented in MS2 to yield a single ion at *m*/*z* 227 [M − H − 162]^−^ (loss of a glucose molecule) [33,36]. The latter stilbene was found only in the grape stems. The peaks **St5** and **St7** corresponded to isomeric forms of Σ-viniferin according to the [M − H]^−^ pseudo molecular ion at *m*/*z* 453 amu which after fragmentation generated in MS2 the product ion *m*/*z* 359 amu corresponding to [M − H − 94]^−^ (loss of a single phenol group). Both isomers were present in the three matrices (grape stems, grape pomace, and wine lees), and thus appear to be the most ubiquitous stilbenes of the winery by-products [14].

Finally, the peak **St4** was identified in the grape stem and pomace as the stilbenoid tetramer known as hopeaphenol (molecular formula C_56_H_42_O_12_) with a [M − H]^−^ pseudo molecular ion at *m*/*z* 905 amu. In turn, the pseudo molecular ion at *m*/*z* 717 amu produced in MS2 is the result of the loss of two phenol groups (94 + 94) [M − H − 188]^−^ [37] and has been previously detected in wine [39].

#### 2.1.4. Flavonols

**Figure 4:**
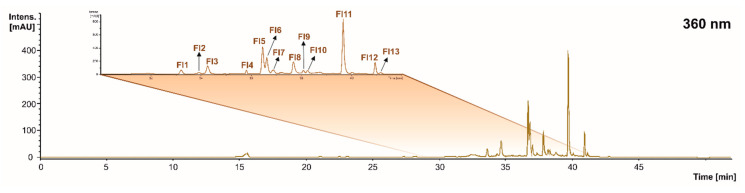
Chromatogram recorded at 360 nm representative of the flavonols (Fl) recorded in grape stems, grape pomace, and wine lees. Identification of the peaks annotated according to Table 4.

When assessing the flavonol profile of the grape stems and pomace, as well as wine lees, 13 compounds were found in concentrations higher than the LOD (Figure 1 and Figure 4 and Table 4). In the same way, the tentative identification performed at 360 nm allowed us to describe an irregular distribution of eleven flavonols in the grape stems, five in the grape pomace, and eleven in the wine lees.

The individual flavonols identified were 3’,5’-di-methyltricetin derivative (peaks **Fl1** and **Fl2**), myricetin hexoside (peak **Fl3**), kaempferol 3-glucoside (peak **Fl4**), quercetin 3-glucuronide (peak **Fl5**), quercetin 3-glucoside isomers (peaks **Fl6** and **Fl7**), myricetin (peak **Fl8**), kaempferol-glucoside (peak **Fl9**), isorhamnetin hexoside (peak **Fl10**), quercetin (peak **Fl11**), kaempferol (peak **Fl12**), and isorhamnetin (peak **Fl13**) (Figure 4 and Table 4).

The flavonols represented by the peaks **Fl1** and **Fl2** were two isomers of a flavonol identified as 3’,5’-di-methyltricetin derivative according to its [M − H]^−^ pseudo molecular ion (*m*/*z* 509 amu). The MS2 fragmentation of these two isomers generated product ions at *m*/*z* 329 amu [M − H − 162 − 18]^−^ (loss of hexoside plus water) [40]. Isomer 1 was found in the grape stems and wine lees, while isomer 2 only appeared in the wine lees. This constitutes additional evidence of the relevance of the vinification process and the capacity of yeast and LAB’s fermentation to set up the actual (poly)phenolic burden of the different residues.

The flavonols **Fl6** and **Fl7** corresponded to two isomeric forms of quercetin 3-glucoside (molecular formula C_21_H_19_O_12_). Both isomers presented a [M − H]^−^ pseudo molecular ion at *m*/*z* 463 amu and exhibited a fragmentation pattern towards the MS2 product ion at *m*/*z* 301 amu [M − H − 162]^−^, which is indicative of a loss of a glucose molecule [33]. **Fl6** was present in all the matrices analyzed, while isomer Fl7 was only found in the grape stems and wine lees. Similarly, the flavonols corresponding to peaks **Fl4** and **Fl9** were identified as kaempferol glucoside isomers (molecular formula C_21_H_20_O_11_) due to the presence of deprotonated parent ions at *m*/*z* 447 amu that fragmented towards a base peak at *m*/*z* 285 amu [M − H − 162]^−^ (loss of a glucose molecule) [38]. Additionally, regarding glucosylated compounds, the peaks **Fl3** and **Fl10** were identified as myricetin hexoside (C_21_H_20_O_13_) and isorhamnetin hexoside (C_22_H_22_O_12_) due to the [M − H]^−^ pseudo molecular ions at *m*/*z* 479 and 477 amu, respectively, which correspond to the major deprotonated fragments in MS2 at *m*/*z* 317 and 315 amu [M − H − 162]^−^ (loss of glucose) [41].

An additional esterified flavonol found in all three winery by-products was quercetin 3-glucuronide (**Fl5**, molecular formula C_21_H_18_O_13_), which presented a parent [M − H]^−^ pseudo molecular ion at *m*/*z* 477amu that by fragmentation gave rise to a major MS2 fragment at *m*/*z* 301 corresponding to unesterified quercetin after the loss of glucuronide moiety [M − H − 176] [38].

Finally, and in good agreement with the recognized lower polarity of unesterified flavonols, myricetin (C_15_H_10_O_8_), quercetin (C_15_H_10_O_7_), kaempferol (C_15_H_10_O_6_), and isorhamnetin (C_16_H_12_O_7_) (peaks, **Fl8**, **Fl11**, **Fl12,** and **Fl13**, respectively) were detected. These flavonols presented [M − H]^−^ pseudo molecular ions at *m*/*z* 317, 301, 285, and 315 amu, respectively. The most abundant product ions in the MS2 spectra for these unesterified flavonols were *m*/*z* 179, 179, 214, and 301 amu, respectively [33,43,44,45]. All of these flavonols, except for the isomers of 3’,5’-di-methyltricetin derivatives, have been recently reported in wine by-products, such as stems, seeds, and pomace [32].

#### 2.1.5. Anthocyanins

**Figure 5:**
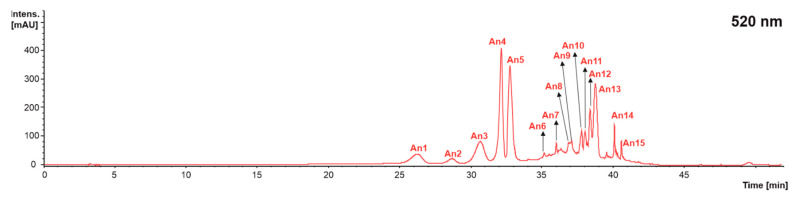
Chromatogram recorded at 520 nm representative of the anthocyanins (An) recorded in grape stems, grape pomace, and wine lees. Identification of the peaks annotated according to Table 5.

When profiling the individual anthocyanins present in the grape stem, grape pomace, and wine lees, 15 individual anthocyanins were identified at 520 nm in the positive mode (Figure 1 and Figure 5 and Table 5). A comparison of the separate by-products allowed us to describe the presence of 13 anthocyanins in the grape stems, 14 in the grape pomace, and 15 in the wine lees.

The individual anthocyanins identified were delphinidin-3-glucoside (**An1**), cyanidin 3-glucoside (**An2**), petunidin 3-glucoside (**An3**), peonidin 3-glucoside (**An4**), malvidin 3-glucoside (**An5**), delphinidin 3-acetylglucoside (**An6**), petunidin 3-acetylglucoside (**An7**), peonidin 3-acetylglucoside (**An8**), malvidin 3-acetylglucoside (**An9**), delphinidin 3-*p*-coumaroylglucoside (**An10**), malvidin 3-6-caffeoyl-glucoside (**An11**), cyanidin 3-*p*-coumaroylglucoside (**An12**), petunidin 3-coumaroylglucoside (**An13**), peonidin 3-(6-*trans*-*p*-coumaroyl)-glucoside (**An14**), and malvidin 3-*p*-coumaroylglucoside (**An15**) (Figure 5 and Table 5). This anthocyanin profile was very similar to those previously described by Souza da Costa et al. (2022) in wine by-products from both conventional and carbonic maceration winemaking [32,46].

Regarding the glycosylated form of the anthocyanins present in grapes residues, it was observed that delphinidin 3-glucoside (C_21_H_21_ClO_12_^+^), cyanidin 3-glucoside (C_21_H_21_ClO_11_), petunidin 3-glucoside (C_22_H_23_ClO_12_), peonidin 3-glucoside (C_22_H_23_O_11_^+^), and malvidin 3-glucoside (C_23_H_25_ClO_12_) (peaks **An1**–**An5**) presented [M]^+^ pseudo molecular ions at *m*/*z* 465, 449, 479, 463, and 493 amu, respectively. The MS2 fragmentation pattern evidenced in all cases the loss of a glucose molecule [M − 162]^+^ to yield protonated ions at *m*/*z* 303, 387, 317, 301, and 331 amu, respectively, which is in good agreement with previous descriptions in the literature of the anthocyanin profile of grape and grape by-products [32,45].

Additionally, acetylglucoside esterifications of delphinidin (**An6**), petunidin (**An7**), peonidin (**An8**), and malvidin (**An9**) were found in concentrations higher than the LOD of the method (molecular formulas C_27_H_31_O_17_^+^, C_24_H_25_O_13_^+^, C_24_H_25_O_12_^+^, C_25_H_27_O_13_^+^, respectively), displaying protonated ([M]^+^) pseudo parent molecular ions at *m*/*z* 507, 521, 505, and 535 amu, respectively, and MS2 major fragments at *m*/*z* 303, 317, 301, and 331 amu due to the loss of the acetylglucoside moiety [M + H − 204]^+^ [45].

Additionally, the presence of malvidin 3,6-caffeoyl-glucoside (**An11**, C_32_H_31_O_15_^+^) was detected, featuring a [M]^+^ pseudo molecular ion at *m*/*z* 665 amu that generated a final MS3 fragment at *m*/*z* 315 amu [M − 350]^+^ corresponding to the loss of the glucose and caffeic acid moieties (162 and 180 amu, respectively) [45], as well as a compound recently described in three varieties of grape pomace (‘Carménère’, ‘Merlot’, and ‘Cabernet Sauvignon’) [47].

Finally, delphinidin 3-*p*-coumaroylglucoside (C_30_H_27_O_14_^+^), cyanidin 3-*p*-coumaroylglucoside (C_30_H_25_O_13_^−^), petunidin 3-*p*-coumaroylglucoside (C_31_H_29_O_14_^+^), peonidin 3-(6-*trans*-*p*-coumaroyl)-glucoside (C_3_H_29_O_13_^+^), and malvidin 3-*p*-coumaroylglucoside (C_32_H_31_O_14_^+^) were also identified on the basis of their [M]^+^ pseudo molecular ions, recorded at *m*/*z* 611, 595, 625, 609, and 639 amu, respectively, and corresponding MS2 fragments at *m*/*z* 303, 287, 317, 301, and 331 amu [M − 326 + 18]^+^ (loss of the coumaroylglucose moiety and acquisition of a water molecule) [45].

### 2.2. Quantitative (Poly)phenolic Content of Grape Stems, Grape Pomace, and Wine Lees

The (poly)phenolic profiles of the grape stems, grape pomace, and wine lees revealed to a great extent a complementary distribution of compounds belonging to the different families of (poly)phenols (catechin derivatives, proanthocyanidins, phenolic acids, stilbenes, flavonols, and anthocyanins). However, despite the common phenolic profile among the three oenology by-products (Figure 1), important quantitative differences were observed among them. In addition, the microbiological burden characterizing each by-product strongly conditioned the phenolic diversity due to the metabolism of the original compounds present in the plant material, as stated by Speranza et al. (2017) [48]. Because of the functional attributes of these compounds, the diversification of the chemical traits of the newly synthesized derivatives could give rise to additional functionalities as a result of new capacities to interact with molecular targets in cells [1]. Additionally, the different profiles generated in each by-product after the metabolization of the original (poly)phenols in the grape stems and pomace, as well as the wine lees, led to envisaging new formulations based on the combination of the three residues, each of them supplying bioactive compounds with specific functionalities, thus contributing to additive mixes or synergies between the separate materials [39]. In this regard, profiling grape pomace and wine lees shed light on the role played by yeasts and LAB (especially in the wine lees, the nowadays less-characterized winery by-product); the modification of their phenolic profiles is more evident because of the yeast and LAB metabolism (Table 1, Table 2, Table 3, Table 4 and Table 5). The resulting description will contribute to identifying the potential of winery by-products for the generation of new ingredients or foods with added value, have a significant impact on human health, and further contribute to a circular economy.

#### 2.2.1. Catechin Derivatives and Proanthocyanidins

**Figure 6:**
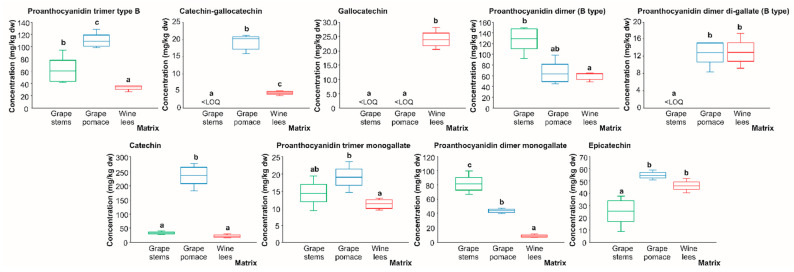
Box plots with quartiles (upper values 75%, median 50%, and lower values 25%) of individual catechin derivatives and proanthocyanidins (mg/kg dw) of (poly)phenolic extracts of grape stems (green box), grape pomace (blue box), and wine lees (red box). Boxes with a different letter (a–c) within each plot indicate statistically significant differences (*p* < 0.05) according to the one-way analysis of variance (ANOVA) and Tukey’s multiple range test. <LOQ, lower than the limit of quantification.

Regarding flavan-3-ol derivatives and proanthocyanidins, although twenty-nine individual compounds were found at a level higher than the LOD allowing for their proper identification, only eight compounds presented concentrations higher than the limit of quantification (LOQ). These were found in the following decreasing order of concentration (average of the concentration recorded in all matrices): catechin (96.15 mg/kg dw) > proanthocyanidin dimer (B-type) (84.60 mg/kg dw) > proanthocyanidin trimer (B-type) (67.67 mg/kg dw) > proanthocyanidin dimer monogallate (45.04 mg/kg dw) > epicatechin (42.02 mg/kg dw) > proanthocyanidin dimer digallate (8.61 mg/kg dw) > gallocatechin (7.99 mg/kg dw) > catechin-gallocatechin (7.83 mg/kg dw) (Figure 6). In general, it is accepted that proanthocyanidins are found in higher concentrations in woody tissues, typical of the nature of the vine [49] and also represent the most relevant phenolic compounds in grape stems [14]. This by-product displayed a higher concentration of proanthocyanidin dimer (B-type) and proanthocyanidin dimmer monogallate (128.88 and 82.19 mg/kg dw, respectively), surpassing the content of proanthocyanidin dimer (B-type) of the grape pomace and wine lees by 50.0%, on average, and the level of proanthocyanidin dimer monogallate of the grape pomace and wine lees by 46.3% and 89.3%, respectively (Figure 6).

On the other hand, proanthocyanidin trimer (B-type), catechin-gallocatechin, and catechin were found at the highest concentration in the grape pomace (109.41, 20.20, and 233.62 mg/kg, respectively), being mainly provided by the grape skin and seeds as previously reported [50]. Additionally, the grape pomace was the by-product exhibiting the highest concentration of proanthocyanidin dimer digallate and epicatechin jointly with the wine lees (12.91 and 58.69 mg/kg dw, on average, respectively). Proanthocyanidin trimer monogallate showed limited statistical differences corresponding to the highest concentration of the grape pomace (19.15 mg/kg dw) which significantly surpassed that of the wine lees by 41.2% (Figure 6), possibly due to the diverse tissues of this by-product [51]. Regarding the lees, the differentiating molecule was gallocatechin (23.97 mg/kg dw), which was almost absent in the grape stems and pomace. Nevertheless, it should be noted that this compound has previously been detected in lyophilized grape pomace and stems, ranging from 380.55 to 1.97 mg/kg dw, respectively [32], thus supporting the fact that the composition of the phenolic compounds of winery residues depends on several factors, including the grape variety, grape maturity, regions of origin, agronomic characterization, ethanol content, fermentation temperature, maceration length, and winemaking techniques applied [52].

When viewing the quantitative flavan-3-ols derivative’s profile as a whole, it is noticed that, interestingly, the grape pomace and wine lees presented high concentrations of monomers of flavan-3-ols in comparison with the grape stems. This fact could be due to the catabolization capacity of yeasts and LAB present in the grape must during the fermentation process. In regards to this matter, to the best of our knowledge, the hydrolysis of proanthocyanidins by yeasts has not been described yet, though LAB species (*Lactiplantibacillus plantarum*) and also colonic microbiota have been shown to provide bioavailable compounds with proven biological benefits in humans [46,53,54]. So, the enzymes responsible for the hydrolysis reaction are a type of esterase known as tannase (tannin acyl hydrolase (EC 3.1.1.20)) that has been broadly applied in the food and pharmaceutical industries [55].

#### 2.2.2. Phenolic Aids

As for flavan-3-ol derivatives, in most of the phenolic acids identified in the grape stems and pomace, as well as the wine lees (*n* = 15, Table 2), the number of compounds found at concentrations higher than the LOQ was closely dependent on the matrix considered (grape stems, 2; grape pomace, 7; and wine lees, 4). In this regard, to date, it has been noted that many of the phenolic acids usually present in grape pomace and wine lees are metabolized by the microorganisms involved in the winemaking process, giving rise to newly synthesized compounds, which, in turn, are responsible for the aromatic composition of wine [56].

**Figure 7:**
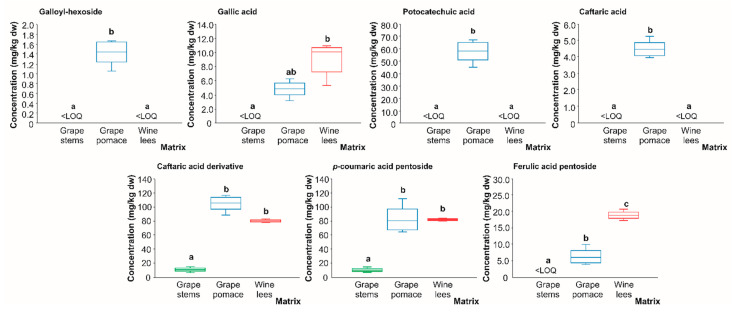
Box plots with quartiles (upper values 75%, median 50%, and lower values 25%) of individual phenolic acids (mg/kg dw) of (poly)phenolic extracts of grape stems (green box), grape pomace (blue box), and wine lees (red box). Boxes with a different letter (a–c) within each plot indicate statistically significant differences (*p* < 0.05) according to the one-way analysis of variance (ANOVA) and Tukey’s multiple range test. <LOQ, lower than the limit of quantification.

Regarding the phenolic acids present in wine by-products in quantifiable concentrations (Figure 7), almost all of them were found at the highest concentration in the grape pomace, except for ferulic acid pentoside, whose highest amount was found in the wine lees. In contrast, the lowest concentration for all of them corresponded to the grape stems which are marked by the outstanding content of proanthocyanidins [57]. In grape pomace and wine lees, the average concentrations of the phenolic acids present in levels higher than the LOQ, galloyl hexoside (only in grape pomace), gallic acid, protocatechuic acid (only in grape pomace), caftaric acid (only in grape pomace), caftaric acid derivative, *p*-coumaric acid pentoside, and ferulic acid pentoside, were found in concentrations above the LOQ in the grape pomace and wine lees (1.44, 7.01, 58.13, 4.45, 92.84, 82.24, and 12.73 mg/kg dw, respectively). To understand the different phenolic compositions of the grape pomace and wine lees relative to the grape stems, it should be noted that both the pomace and lees during the winemaking process are in contact with the yeasts and LAB. In this sense, Teixeira et al. (2014) reported that yeasts possess enzymes responsible for the hydrolysis and transformation of complex (poly)phenolic substrates into phenolic compounds with a high added value [57], such as gallic acid, whose highest concentration is in wine lees (Figure 6).

These molecular transformations are relevant because they provide those matrices with specific (poly)phenolic profiles featuring high concentrations of strongly antioxidant compounds. Moreover, the grape pomace was the only plant material that obtained quantifiable concentrations of galloyl-hexoside, protocatechuic acid, and caftaric acid, and had a protocatechuic mean concentration of 58.13 mg/kg dw. Again, as for the wine lees, the grape pomace is involved in the winemaking process, in contrast to grape stems, so it could be also subject to the fermentation process and the consequent transformation of the phenolic burden by yeast and LAB. As a result of such metabolization, phenolic diversity is obtained with a positive impact on biology (bioavailability and bioactivity) [58]. In this sense, particularly gallic acid and protocatechuic acid can be decarboxylated to pyrogallol and catechol, respectively, by cultures of *L. plantarum*, a malolactic starter culture in winemaking [59]. Nonetheless, as both grape pomace and wine lees are exposed to the fermentation process, the different profiles obtained afterwards may suggest that the phenolic acids found only in grape pomace could have not been transformed in the same way (at least as the unique source), as in wine lees to some phytochemicals.

Finally, in regards to phenolic acids, ferulic acid pentoside, although at a low concentration, is mainly present in a significant way in the wine lees. The possible transformation that takes place in the wine lees could also be observed in the data for ferulic acid pentoside in which it appears to be the majority.

#### 2.2.3. Stilbenes

**Figure 8:**
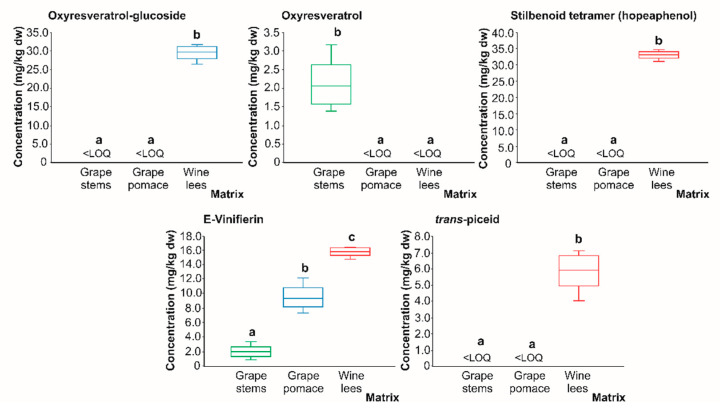
Box plots with quartiles (upper values 75%, median 50%, and lower values 25%) of individual stilbenes (mg/kg dw) of (poly)phenolic extracts of grape stems (green box), grape pomace (blue box), and wine lees (red box). Boxes with a different letter (a–c) within each plot indicate statistically significant differences (*p* < 0.05) according to the one-way analysis of variance (ANOVA) and Tukey’s multiple range test. <LOQ, lower than the limit of quantification.

In regards to stilbenes (Figure 8), oxyresveratrol-glucoside, stilbenoid tetramer, and *trans* piceid acid were found only in the wine lees in quantifiable concentrations (29.55, 33.03, and 5.92 mg/kg dw, on average, respectively). Thereby, these phenolics could be considered bioactive compounds characteristic of wine lees, resulting from the metabolism of grape (poly)phenols during the fermentation process. In this regard, it would be important to mention that, currently, it is known that the elicitation of in vitro plant cultures with yeasts improves the biosynthesis of stilbenes (resveratrol and oxyresveratrol); thus, the main results obtained in the present work are an additional demonstration of the synthesis of stilbenoids in wine and winemaking residues by yeasts and LAB [60].

These findings would support the contribution of these microorganisms to the diversification of the bioactive compounds present in winery by-products, as well as to the formation of the derivatives of said molecules. This would entail an enhancement of our biological scope because of the possible interaction of the new phenolics formed with molecular targets responsible for complementary forms of modulating cell biology. In the case of oxyresveratrol (Figure 8), low concentrations were found only in the grape stems (2.12 mg/kg dw). This finding, together with the formation of oxyresveratrol-glucoside in the wine lees, may indicate the rapid metabolism of oxyresveratrol into glucosylated derivatives by yeasts [60]. Regarding E-Viniferin, this *trans* resveratrol dimer was found in all three residues analyzed in the following decreasing order: wine lees (15.79 mg/kg dw) > grape pomace (9.47 mg/kg dw) > grape stems (1.96 mg/kg dw). Again, the highest concentration recorded for E-Viniferin in the wine lees is possibly a result of hydrolysis reactions of more complex stilbenes by yeast and LAB [58]. The formation of E-Viniferin could be useful in the thin preparation of future functional foods or supplements that could contribute to the prevention and/or treatment of chronic diseases in humans due to its outstanding anti-inflammatory properties and oxidative stress [60]. This molecule is known to be produced in vitro by the oxidative dimerization of resveratrol by plant peroxidases or by fungal laccases, which reinforces the hypothesis of the intervention of fermenting microorganisms [61].

#### 2.2.4. Flavonols

**Figure 9:**
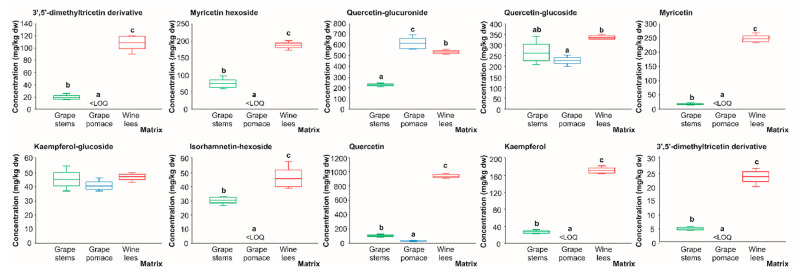
Box plots with quartiles (upper values 75%, median 50%, and lower values 25%) of individual flavonols (mg/kg dw) of (poly)phenolic extracts of grape stems (green box), grape pomace (blue box), and wine lees (red box). Boxes with a different letter (a–c) within each plot indicate statistically significant differences (*p* < 0.05) according to the analysis of variance (ANOVA) and Tukey’s multiple range test. <LOQ, lower than the limit of quantification.

Regarding flavonols, which are mainly located in the skin of grapes, as well as, to a lesser extent, in the seeds of some varieties [62], seven out of the ten compounds with concentrations higher than the LOQ (3’-5’-di-methyltricetin derivative, myricetin hexoside, myricetin, isorhamnetin-hexoside, quercetin, and kaempferol) were found to have their highest concentration in the wine lees (109.10, 187.00, 247.30, 45.30, 934.85, and 170.69 mg/kg dw, respectively) (Figure 9). On the other hand, quercetin-glucuronide, the second major flavonol after quercetin in the samples analyzed, appeared at the highest level in the grape pomace (611.57 mg/kg dw), which significantly surpassed its levels in the wine lees and grape stems by 13.4 and 63.0%, respectively (Figure 9). These findings are in line with a recent study of winery by-products in which quercetin-glucuronide was the most abundant among all the quercetin derivatives detected in grape pomace [32].

Regarding quercetin glucoside, the concentrations recorded (335.01, 263.95 and 227.25 mg/kg dw) were also noticeable in the wine lees, grape stems, and grape pomace, respectively, appearing to only have statistically significant differences between the wine lees and grape pomace (*p* < 0.05). These results were very similar to the recently reported results by Souza da Costa, et al. 2022, in which the authors found 261.41 and 116.34 mg/kg dw of quercetin glucoside in grape stems and pomace, respectively [32]. To a lesser extent, kaempferol glucoside was present in almost equal concentrations in all three of the matrices analyzed (44.17 mg/kg dw on average in all matrices) (Figure 9), a finding that follows a recent study in which kaempferol glucoside was detected from 62.61 to 25.66 mg/kg dw in grape stems and pomace, respectively [32].

In summary, these results evidenced that, in general, the wine lees were the residues with the highest flavonol burden (except for quercetin 3-glucuronide, which displayed the highest concentration in grape pomace). Thereby, they should be considered a valuable foodstuff for the generation of new functional ingredients, foods, or feeds, providing a diversity of flavonols with beneficial effects for health (e.g., having an antihypertensive capacity; acting in psychiatric disorders by having psychostimulant effects; having a neuroprotective effect in Parkinson’s disease; having antimicrobial, antifungal, and antiviral functions; being used for metabolic disorders; having a wide variety of anticancer effects; and providing antioxidant and anti-inflammatory activities) [63,64,65,66]). On the other hand, grape stems were identified also as an interesting source of flavonols according to their quantity and diversity.

The autolysis of the yeasts in the wine lees [67], in addition to their transformation as a result of the interaction with the bioactive molecules, with which yeasts have an active interaction [68], could lead to the modification of their phytochemical profile with great potential. Even though enological strategies have been developed to promote improvements in quality, the exploitation of phenolic compounds present in wine and wine by-products continues to be an underexplored challenge; notably, it would contribute to the development of new winery by-product-based functional ingredients, especially for wine lees when talking about flavonols. As discussed previously, the broad diversity of flavonols (as well as other phenolics) in this matrix seems to be associated with the metabolism of yeasts and LAB.

#### 2.2.5. Anthocyanins

Along with flavonols, the presence of compounds belonging to the anthocyanin phenolic class has been well-documented in wine and winery by-products [48,56]. So, the present study contributes this characterization, expanding the spectrum of matrices already assessed to other by-product matrices that have been underexplored to date.

**Figure 10:**
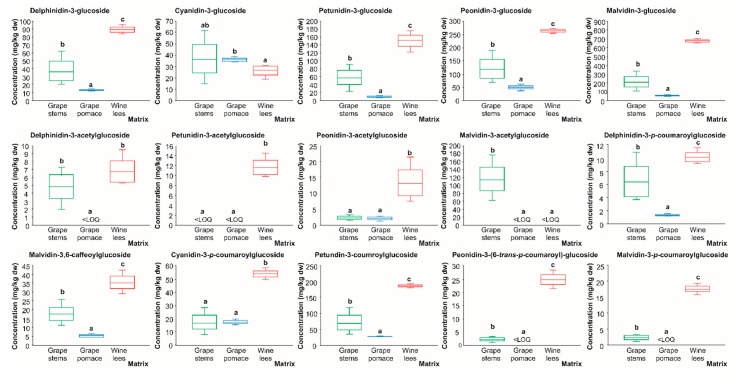
Box plots with quartiles (upper values 75%, median 50%, and lower values 25%) of individual anthocyanins (mg/kg dw) of (poly)phenolic extracts of grape stems (green box), grape pomace (blue box), and wine lees (red box). Boxes with a different letter (a–c) within each plot indicate statistically significant differences according to the one-way analysis of variance (ANOVA) and Tukey’s multiple range test. <LOQ, lower than the limit of quantification.

The results retrieved evidenced that the 3-glucoside derivatives of delphinidin, petunidin, peonidin, and malvidin were found at the highest concentration in the wine lees, which exhibited concentrations ranging between 89.29 and 670.47 mg/kg dw (Figure 10). The second residue that was a source of 3-glucose anthocyanins was grape stems (36.7, 57.30, 120.42, and 213.11 mg/kg dw, respectively). In terms of malvidin 3-glucoside, the anthocyanin with the highest concentration in the three matrices assessed, our findings were in line with a recent study performed on grape by-products [32].

Regarding anthocyanins’ acetylglucoside derivatives, the highest amounts corresponded again to the wine lees with values ranging between 6.71 and 13.23 mg/kg dw, except for malvidin-3-acetylglucoside, which was only found in quantifiable concentrations in the grape stems (2.01 mg/kg dw), and delphinidin 3-acetylglucoside, which had no significant differences between the grape stems and wine lees (5.77 mg/kg dw, on average), while it was under the LOQ in the grape pomace (Figure 10).

Similarly to the previously referenced metabolites of grape anthocyanins, delphinidin-3-*p*-coumaroylglucoside, malvidin 3,6-caffeoylglucoside, cyanidin 3-*p*-oumaroylglucoside, petunidin 3-coumaroylglucoside, peonidin 3-(6-*trans*-*p*-coumaroyl)-glucoside, and malvidin 3-*p*-coumaroylglucoside were found at the highest concentrations in the wine lees (10.04, 35.22, 53.14, 186.47, 24.55, and 17.54 mg/kg dw) (Figure 10).

Although the mechanisms responsible for such diversity are poorly understood (even more so in regards to winery by-products), the fermentation process’s impact on the diverse composition of phytochemicals is well known [57] and this should be considered when considering the by-products obtained after the fermentation process, which are in contact with fermenting microorganisms. Thereby, once again, the differential profile of the wine lees (in this case, in terms of anthocyanins) puts a spotlight on the relevance of LAB and yeast metabolism in increasing and transforming (poly)phenolic profiles [58,60], giving rise to a broader amount and diversity. In the same vein, previous studies have displayed that LAB (*Oenococcus oeni* and *Lactiplantibacillus plantarum*) strains can absorb anthocyanin glucosides through the cell wall, through the production of β-glycosidase enzymes that cleave the anthocyanin glucoside glycosidic bonds and can further degrade the aglycons into phloroglucinol aldehyde and corresponding phenolic acids [69,70]. In this way, the absorption rate of anthocyanin glucosides, the β-glycosidase activity, and the degradation rate of the anthocyanins are dependent on the species and strains of LAB and yeasts [70].

In summary, regarding the quantitative profile of wine by-products (grape stems, pomace, and wine lees), proanthocyanidins (dimer (B-type)) were the more abundant phenolic compounds in the grape stems, phenolic acids (protocatechuic and caftaric acids) in the grape pomace, and flavonols (except quercetin-glucuronide, which has a majority in the grape pomace) and anthocyanins (mainly, malvidin-3-glucoside) in the wine lees. These findings agree with those of a previous report [71]. Thus, the exploitation of these wine by-products with particular profiles could be of special interest for screening the synergies among them to develop new ingredients with relevant biological properties.

### 2.3. Differential DPPH^●^ Scavenging Activity and Correlation Analysis

**Figure 11:**
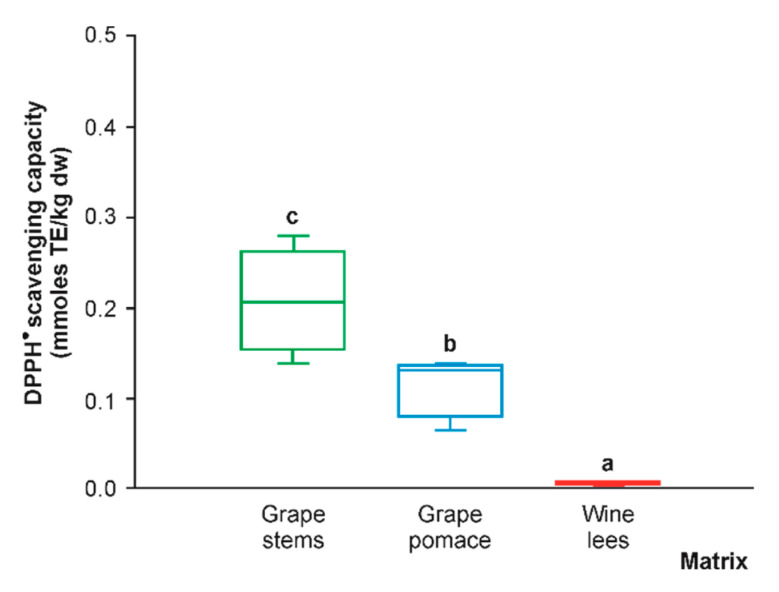
Box plots with quartiles (upper values 75%, median 50%, and lower values 25%) of DPPH^●^ scavenging capacity (mmol TE/kg dw) of (poly)phenolic extracts of grape stems (green box), grape pomace (blue box), and wine lees (red box). Boxes with a different letter (a–c) within each plot indicate statistically significant differences according to the one-way analysis of variance (ANOVA) and Tukey’s multiple range test.

Because of the broad information included in the article on the (poly)phenolic composition of the grape stems, grape pomace, and wine lees, the radical scavenging capacity of these materials was assessed by a single method. Because of the accepted requirement to measure antioxidant activity by eight or more methods, we selected the DPPH test, according to Baliyan et al. (2022), since this method is the quickest, easiest, and most affordable approach for the measurement of antioxidant power [72]. When evaluating the DPPH^•^ scavenging capacity of the hydro-methanolic extracts of phenolic compounds of the grape stems, grape pomace, and wine lees, the significantly highest values corresponded to the grape stems (183.55 mmol TE/kg dw, on average), which surpassed the anti-radical power of the grape pomace and wine lees by 47.3% and 98.6%, respectively (Figure 11). Recently, the phenolic extracts obtained from grape stems have been gaining more attention as antioxidants [73,74,75].

The analysis of correlation pointed out significant positive associations of individual phenolic compounds in the following order of decreasing regression strength according to the R^2^ coefficient, proanthocyanidin dimer monogallate (0.744; *p* < 0.01) > oxyresveratrol (0.603; *p* < 0.05).

Additionally, since the present work was focused on the characterization of hydro-methanolic extracts, the results obtained can be considered in designing winery by-products as sources of bioactive (poly)phenols for applications other than as a dietary antioxidant supplement (e.g., cosmetic or technological uses). However, additional determinations of the bioaccessibility of phenolic compounds from the separate matrices and the antioxidant activity of the digested fractions should be developed to identify the best source of dietary antioxidants [57].

## 3. Materials and Methods

### 3.1. Chemicals and Reagents

The compounds 2,2-diphenyl-1-picrylhydrazyl radical (DPPH^•^) as well as the standard catechin, 1,4-chlorogenic acid, resveratrol, quercetin-3-*O*-glucoside, and cyanidin-3-*O*-glucoside were obtained from Sigma-Aldrich (Steinheim, Germany). The 6-hydroxy-2,5,7,8-tetramethylchroman-2-carboxylic acid (Trolox) was purchased from Fluka Chemika (Neu-Ulm, Switzerland). Formic acid was purchased from Panreac (Castellar del Vallés, Barcelona, Spain). All LC-MS grade solvents were obtained from JT Baker (Phillisburg, NJ, USA). Ultrapure water was produced using a Millipore water purification system.

### 3.2. Plant Material

Solid (grape (*Vitis vinifera* L. var. ‘Monastrell’) stems and pomace) and semisolid (wine lees) winery by-products were obtained from the winery Industry Bodegas Viña Elena S.L. (Jumilla, Murcia, Spain) during the 2020 season. For analytical purposes, these by-products were freeze-dried up to constant weight using a CHRIST vacuum concentrator 2-4D (Wolflabs, York, UK). The dry materials were ground to a fine powder, stored, and protected from light for further phytochemical and radical scavenging analyses.

### 3.3. Qualitative and Quantitative (Poly)phenolic Profile by HPLC-PDA-ESI-MSn

Dehydrated samples (100 mg) were mixed with 1 mL of methanol/formic acid/water (50:2:48, *v*/*v*/*v*), vortexed, and sonicated in an ultrasonic bath for 60 min. Afterwards, the samples were kept at 4 °C overnight and sonicated again for 60 min. Then, a centrifugation step at 10,000× *g* for 5 min was used to separate the extract from the solid residue, and the supernatants were filtered through a 0.22 µm PVDF filter (Millex HV13, Millipore, Bedford, MA, USA).

The chromatographic separation of the phenolic compounds present in the analytical extracts was performed on a Luna C18 column (250.0 × 4.6 mm, 5.0 µm particle size, Phenomenex, Macclesfield, UK) using an Agilent HPLC 1100 series equipped with a diode array detector and mass detector in series (Agilent Technologies, Waldbronn, Germany). The HPLC consisted of a binary pump (model G1312A), an autosampler (model G1313A), a degasser (model G1322A), a photodiode array (PDA) detector (model G1315B), and an ion trap spectrometer (model G2445A) equipped with an electrospray ionization interface and controlled by LCMSD software (v. 4.1, Agilent Technologies), operated according to the chromatographic and ionization specifications described by Barros et al. [14]. The mobile phases used were deionized water/formic acid (99.0:1.0, *v*/*v*) (solvent A) and acetonitrile/formic acid (9.0:1.0, *v*/*v*) (solvent B). Spectral data from all peaks were detected in the 200–600 nm range and chromatograms were recorded at 280 nm for proanthocyanidins, 330 nm for phenolic acids and stilbenes, 360 nm flavonols, and 520 nm for anthocyanins. The HPLC-PDA-ESI/MSn analyses were carried out by the ChemStation software (v. 08.03, Agilent Technologies). Mass spectrometry data were acquired in the negative (proanthocyanidins, phenolic acids, stilbenes, and flavonols) and positive (anthocyanins) ionization modes. The identification of the phenolic compounds was performed by examining the retention time (min), parent ions, and the MS2 and MS3 fragmentation patterns in comparison with those of databases and descriptions available in the literature. Phenolic compounds were characterized and quantified by PDA chromatograms recorded at the different wavelengths described above for the distinct phenolic classes, using daily prepared calibration curves, with catechin (proanthocyanidins), chlorogenic acid (phenolic acids), resveratrol (stilbenes), quercetin-3-*O*-glucoside (flavonols), and cyanidin-3-*O*-glucoside (anthocyanins).

### 3.4. Antioxidant Capacity

The free radical scavenging activity was determined by the DPPH^●^ method adapted to microscale according to Domínguez-Perles et al. [9], measuring the variation in the absorbance of the DPPH^●^ solution at 515 nm after 50 min of reaction. The results were expressed as mM of Trolox equivalents per g of dry weight (mM TE/g dw).

### 3.5. Statistical Analysis

The design of the experiment was completely randomized with six replications (*n* = 6) for each plant material (grape stem, grape pomace, and wine lees). Results are presented in bar plots with an indication of the mean ± SD. Data were analyzed using the Statistical Package for the Social Sciences (SPSS) 24.0 software package (LEAD Technologies, Inc., Chicago, IL, USA). All data were subjected to a one-way analysis of variance (ANOVA). The fulfilment of the one-way ANOVA requirements, specifically the normal distribution of the residuals and the homogeneity of variance, was tested using the Kolmogorov–Smirnov (with Lilliefors correction) and Levene tests, respectively. When statistical differences were identified, the variables were compared using Tukey’s multiple range test. Pearson’s correlation analysis was performed between the content of phenolic compounds and the radical scavenging capacity to corroborate the relationships between parameters. All statistical tests were performed at a 5.0% significance level.

## 4. Conclusions

The present work constitutes an update of the quantitative phytochemical profile of winery by-products in terms (poly)phenols and their radical scavenging capacity. Thus, it combines wine lees, grape pomace, and grape stems in a detailed test. By extracting the relevance of each one of the by-products in terms of the molecules that it could contribute and its richness, as well as by examining the complementarity of the three matrices (given that they have numerous compounds in common), the present diversification is remarkable. We can greatly expand the range of phytochemicals that can be provided for bioavailability purposes and thereby take advantage of their bioactivity traits. According to the demonstrated benefits of grapes and wine, it would be reasonable to reuse these by-products, as they have similar profiles and potential. As it is pertinent to identify new uses for winery by-products, this would allow us to avoid management costs and enable their valorization as new ingredients, or sources of valuable bioactive nutrients and non-nutrients. Thus, we can provide a market opportunity to develop a more efficient industry, or to generate resources from discarded waste. From this research, we can conclude that in addition to the different compounds associated with the diverse nature of each by-product and its processing specificities, the microorganisms responsible for the fermentation processes associated with wine production are key for the diversification of the phytochemicals present in these materials (especially grape pomace and wine lees) and, therefore, the design of the valorization processes. These are materials containing molecules that can be used in applications based on their valuable technological properties and as health-promoting agents for humans. Furthermore, the different profiles displayed by the separate winery by-products assessed in the present work allow us to envisage their combination to obtain a mixture with the optimal (poly)phenolic burden and thus, take advantage of their application as a technological ingredient or dietary supplement.

## Figures and Tables

**Table 1:** Identified catechin derivatives and proanthocyanidins in grape (*Vitis vinifera* L. var. ‘Monastrell’) stem, pomace, and wine lees by HPLC-PDA-ESI-MSn operating in negative mode.

Peak	Compound	Rt * (min)	*m*/*z* [M − H]	*m*/*z* MS2 [M − H]^−^	*m*/*z* MS3 [M − H]^−^	Ref.	Grape Stem	Grape Pomace	Wine Lees
P1	Proanthocyanidin trimer (B type) isomer 1	7.9	865	695(100), 405(36), 287(29), 577(27)	**695:** 543(100), 391(46), 256(40)	[23,24]	X	X	<LOD
P2	Proanthocyanidin trimer (B type) isomer 2	8.4	865	695(100), 405(36), 287(29), 577(27)	**695:** 543(100), 391(46), 256(40)	[23,24]	X	X	X
P3	Catechin-Gallocatechin isomer 1	8.8	593	441(100), 423(15), 407(12)	**441:** 315(100), 289(52), 153(27), 191(25)	[23,25]	X	X	X
P4	Catechin-Gallocatechin isomer 2	10.7	593	441(100), 423(15), 407(12)	**441:** 315(100), 289(52), 153(27), 191(25)	[23,25]	X	X	X
P5	Gallocatechin	12.0	305	179(100), 219(75), 260(30),	**179:** 167(100), 151(46), 137(11)	[25]	<LOD	<LOD	X
P6	Catechin-Gallocatechin isomer 3	12.5	593	423(100), 305(43), 441(23)	**423:** 283(100), 297(83), 255(25)	[23,25]	<LOD	X	<LOD
P7	Catechin-Gallocatechin isomer 4	13.3	593	423(100), 305(38), 441(25)	**423:** 283(100), 297(60), 255(22)	[23,25]	<LOD	<LOD	X
P8	Procyanthocyanidin dimer (B type) isomer 1	14.3	577	425(100), 407(97), 289(29), 451(16)	**425:** 407(100), 272(10)	[23,24]	X	X	<LOD
P9	Procyanthocyanidin dimer (B type) isomer 2	14.9	577	425(100), 407(90), 289(33), 451(23), 559(17)	**425:** 407(100), 272(10)	[23,24]	X	X	<LOD
P10	Procyanthocyanidin dimer (B type) isomer 3	15.5	577	425(100), 407(97), 289(29), 451(16)	**425:** 407(100), 272(10)	[23,24]	X	X	<LOD
P11	Procyanthocyanidin dimer (B type) isomer 4	16.1	577	407(100), 287(62), 425(54)	**425:** 285(100), 281(59)	[23,24]	X	X	X
P12	Procyanthocyanidin dimer (B type) isomer 5	17.3	577	425(100), 407(71), 289(45), 451(26)	**425:** 407(100), 272(6)	[23,24]	X	X	X
P13	Proanthocyanidin trimer (B type) isomer 3	17.5	865	695(100), 407(43), 287(22), 577(53)	**695:** 543(100), 242(80), 525(66), 405(57)	[23,24]	X	X	X
P14	Proanthocyanidin dimer di-gallate (B type)	17.7	881	695(100), 289(16), 443(16)	**695:** 543(100), 289(32), 242(24)	[23,25]	<LOD	X	X
P15	Proanthocyanidin trimer (B type) isomer 4	17.9	865	695(100), 577(75), 407(63), 303(70), 287(56)	**695:** 405(100), 242(99), 543(87), 677(70), 525(75)	[23,24]	X	<LOD	<LOD
P16	Catechin	18.5	289	245(100), 205(31)	**245:** 202(100), 226(27), 187(22), 161(15)	[24]	X	X	X
P17	Proanthocyanidin trimer (B type) isomer 5	19.2	865	407(100), 577(75), 407(63), 287(56)	**695:** 405(100), 695(83), 577(62), 287(53)	[23,24]	X	X	<LOD
P18	Proanthocyanidin trimer (B type) isomer 6	19.6	865	407(100), 577(75), 407(63), 287(56)	**695:** 405(100), 695(83), 577(62), 287(53)	[23,24]	X	X	X
P19	Proanthocyanidin trimer monogallate isomer 1	20.0	1017	729(100), 677(36), 577(34), 407(19)	**729:** 577(100), 407(56), 451(29), 425(27)	[26,27]	X	<LOD	<LOD
P20	Proanthocyanidin trimer monogallate isomer 2	20.6	1017	729(100), 677(36), 577(34), 407(19)	**729:** 577(100), 407(56), 451(29), 425(27)	[26,27]	X	X	X
P21	Procyanthocyanidin dimer (B type) isomer 6	21.2	577	425(100), 407(83), 289(37), 451(23)	**425:** 407(100), 272(7)	[23,24]	X	<LOD	X
P22	Procyanthocyanidin dimer (B type) isomer 7	21.6	577	425(100), 407(83), 289(37), 451(23)	**425:** 407(100), 272(7)	[23,24]	X	X	X
P23	Proanthocyanidin dimer monogallate isomer 1	22.3	729	577(100), 407(83), 559(62), 425(54), 451(21)	**577:** 407(100), 451(70), 289(29), 425(35)	[27,28]	X	<LOD	<LOD
P24	Proanthocyanidin dimer monogallate isomer 2	23.1	729	407(100), 577(36), 559(31), 451(7)	**407:** 285(100), 257(38), 297(33), 243 (28)	[27,28]	X	X	<LOD
P25	Proanthocyanidin trimer (B type) isomer 7	23.5	865	577(100), 407(80), 695(74), 451(42), 287(42)	**695:** 242(100), 543(90), 407(623), 451(62), 525(57), 289(28)	[23,24]	X	X	X
P26	Epicatechin-glucoside	24.2	449	287(100), 269(60)	**287:** 259(100), 242(10)	[25]	X	<LOD	<LOD
P27	Epicatechin	24.8	289	245(100), 205(29)	**245:** 202(100), 226(29), 187(23), 161(15)	[24]	X	X	X
P28	Proanthocyanidin dimer monogallate isomer 3	25.2	729	407(100), 559(53), 441(39), 577(37), 451(29), 289(29)	**577:** 407(100), 451(70), 289(29), 425(35)	[27,28]	X	X	X
P29	Procyanthocyanidin dimer (B type) isomer 8	25.9	577	425(100), 407(76), 289(32), 451(32)	**425:** 407(100), 272(8)	[23,24]	X	<LOD	X

* LOD, limit of detection; Rt, retention time.

**Table 2:** Identification of phenolic acids in grape (*Vitis vinifera* L. var. ’Monastrell’) stem, pomace, and wine lees by HPLC-PDA-ESI-MSn operating in negative mode.

Peak	Compound	Rt * (min)	*m*/*z* [M − H]	*m*/*z* MS2 [M − H]	*m*/*z* MS3 [M − H]	Ref.	Grape Stem	Grape Pomace	Wine Lees
PA1	Galloyl-hexoside isomer 1	6.2	331	169(100), 271(40), 193(39), 151(269	**169:** 125(100), 151(20)	[24,27]	X	X	<LOD
PA2	Gallic acid isomer 1	7.0	169	125(100)	N.d.	[28,30]	X	X	X
PA3	Gallic acid isomer 2	7.5	169	125(100)	N.d.	[28,30]	X	X	X
PA4	Gentisic acid	9.7	153	153(100)	**153:** 123(100), 109(34)	[30]	X	<LOD	<LOD
PA5	Protocatecuic acid-*O*-hexoside isomer 1	10.4	315	153(100), 165(37), 108(19)	**153:** 108(100), 123(35)	[30]	<LOD	X	<LOD
PA6	Protocatecuic acid-*O*-hexoside isomer 2	11.2	315	153(100), 108(33), 165(15)	**153:** 108(100), 123(35)	[30]	X	X	<LOD
PA7	Galloyl-hexoside isomer 2	11.7	331	169(100), 125(7)	**169:** 125(100)	[24,27]	X	<LOD	<LOD
PA8	Protocatecuic acid-*O*-hexoside isomer 3	12.3	315	153(100), 165(24), 108(10)	**153:** 123(100)	[30]	X	<LOD	<LOD
PA9	Caftaric acid	12.8	311	149(100), 179(38), 135(8)	**149:** 103(100), 131(82)	[30]	<LOD	<LOD	X
PA10	Caftaric acid-glucuronide	14.1	487	355(100), 311(65), 167(42), 211(13)	**355:** 167(100), 311(79), 211(26)	[30]	<LOD	<LOD	X
PA11	Caftaric acid derivative	15.4	623	311(100), 179(7)	**311:** 149(100), 179(51)	[30]	<LOD	<LOD	X
PA12	*p*-coumaric acid pentoside	19.1	295	163(100), 119(8)	**163:** 119(100)	[30]	<LOD	<LOD	X
PA13	*p*-coumaric acid	20.4	162	119(100)	N.d.	[30]	<LOD	<LOD	X
PA14	Ferulic acid pentoside	22.1	325	193(100), 148(4)	**193:** 178(100), 134(84), 148(75)	[30]	<LOD	<LOD	X
PA15	Ethyl gallate	26.1	197	169(100), 125(30)	**169:** 125(100)	[31,32]	X	<LOD	X

* LOD, limit of detection; N.d., not detected; Rt, retention time.

**Table 3:** Identification of stilbenes in grape (*Vitis vinifera* L. var. ’Monastrell’) stem and pomace, as well as wine lees by HPLC-PDA-ESI-MSn operating in negative mode.

Peak	Compound	Rt * (min)	*m*/*z* [M − H]	*m*/*z* MS2 [M − H]	*m*/*z* MS3 [M − H]	Ref.	Grape Stem	Grape Pomace	Wine Lees
St1	Oxyresveratrol-glucoside	27.4	405	243(100)	**243:** 224(100), 198(396), 174(15)	[35]	<LOD	<LOD	X
St2	*Trans*-Piceid isomer 1	32.6	389	227 (100)	**227:** 184(100), 156(37)	[33,36]	X	<LOD	<LOD
St3	Oxyresveratrol	33.5	243	224(100), 198(80), 154(69)	**224:** 180(100), 137(39), 163(14)	[35]	<LOD	<LOD	X
St4	Stilbenoid tetramer (Hopeaphenol)	34.4	905	717(100), 811(94), 359(20), 451(18)	**717:** 675(100), 611(66), 357(53)	[37]	X	X	<LOD
St5	Σ-viniferin isomer 1	35.1	453	359(100), 227(45), 265(23)	**359:** 265(100)	[38]	X	X	X
St6	*Trans*-piceid isomer 2	36.2	389	227(100)	**227:** 184(100), 156(37)	[33,36]	<LOD	<LOD	X
St7	E-viniferin isomer 2	40.2	453	359(100), 227(45), 265(26)	**359:** 265(100)	[38]	X	X	X

* LOD, limit of detection; Rt, retention time.

**Table 4:** Identification of flavonols in grape (*Vitis vinifera* L. var. ’Monastrell’) stem, pomace, and wine lees by HPLC-PDA-ESI-MSn operating in negative mode.

Peak	Compound	Rt * (min)	*m*/*z* [M − H]	*m*/*z* MS2 [M − H]	*m*/*z* MS3 [M − H]	Ref.	Grape Stem	Grape Pomace	Wine Lees
Fl1	3’,5’-di-methyltricetin derivative isomer 1	33.2	509	329(100), 347(61), 355(32), 193(12)	**329:** 313(100), 148(4)	[40]	X	<LOD	X
Fl2	3’,5’-di-methyltricetin derivative isomer 2	33.9	509	329(100), 347(61), 355(32), 193(12)	**329:** 313(100), 148(4)	[40]	<LOD	<LOD	X
Fl3	Myricetin hexoside	34.4	479	317(100), 271(9), 179(4)	**317:** 271(100), 287(36), 179(22), 151(16)	[41]	X	X	X
Fl4	Kaempferol glucoside isomer 1	35.6	447	285(100), 303(53), 151(15), 179(6)	**285:** 241(100), 175(47)	[38]	X	X	<LOD
Fl5	Quercetin 3-glucuronide	36.8	477	301(100), 151(2)	**301:** 151(100), 179(93), 257(34), 272(18)	[38]	X	X	X
Fl6	Quercetin 3-glucoside isomer 1	36.6	463	301(100), 271(7), 343(4), 151(4)	**301:** 179(100), 151(93), 271(38), 255(34)	[33]	X	X	X
Fl7	Quercetin 3-glucoside isomer 2	36.9	463	301(100), 271(7), 343(4), 151(4)	**301:** 179(100), 151(93), 271(38), 255(34)	[33]	X	<LOD	X
Fl8	Myricetin	37.6	317	179(100), 151(60), 193(21)	**179:** 150(100), 169(16)	[42]	X	<LOD	X
Fl9	Kaempferol glucoside isomer 2	38.2	447	285(100), 255(53), 227(12), 169(4)	**284:** 255(100), 227(16), 163(3)	[33]	X	X	X
Fl10	Isorhamnetin hexoside	38.4	477	315(100), 28520), 270(17), 357(15)	**315:** 285(100), 270(71), 299(53), 242(23)	[41]	X	<LOD	<LOD
Fl11	Quercetin	39.7	301	179(100), 151(84), 272(25)	**179:** 151(100), 169(4), 107(4)	[33]	X	<LOD	X
Fl12	Kaempferol	40.9	285	214(100), 153(67), 185(67), 165(52), 257(46)	N.d.	[43]	X	<LOD	X
Fl13	Isorhamnetin	41.2	315	301(100)	**301:** 151(100), 271(70), 227(38), 192(26), 282(27), 164(12)	[44]	<LOD	<LOD	X

* LOD, limit of detection; N.d., not detected; Rt, retention time.

**Table 5:** Identification of anthocyanins in grape (*Vitis vinifera* L. var. ’Monastrell’) stem and pomace, as well as wine lees by HPLC-PDA-ESI-MSn operating in positive mode.

Peak	Compound	Rt* (min)	*m*/*z* [M + H]	*m*/*z* MS2 [M + H]	*m*/*z* MS3 [M + H]	Ref.	Grape Stem	Grape Pomace	Wine Lees
An1	Delphinidin 3-glucoside	26.3	465	303(100)	**303:** 257(100), 229(37), 247(114), 179(13), 275(12)	[45]	X	X	X
An2	Cyanidin 3-glucoside	28.6	449	287(100)	**287:** 193(100), 231(34), 270(30), 109(27), 137(22), 203(16)	[45]	X	X	X
An3	Petunidin 3-glucoside	30.6	479	317(100), 302(1)	**317:** 302(100), 274(39)	[45]	X	X	X
An4	Peonidin 3-glucoside	32.9	463	301(100)	**301:** 286(100), 257(4), 241(2), 230(2)	[45]	X	X	X
An5	Malvidin 3-glucoside	33.1	493	331(100), 316(1)	**331:** 299(100), 315(52), 270(21), 242(17), 179(12)	[45]	X	X	X
An6	Delphinidin 3-acetylglucoside	35.4	507	303(100)	**303:** 257(100), 246(7)	[45]	<LOD	<LOD	X
An7	Petunidin 3-acetylglucoside	36.1	521	317(100)	**317:** 302(100), 274(12)	[45]	<LOD	X	X
An8	Peonidin 3-acetylglucoside	37.0	505	301(100), 286(8)	**301:** 286(100), 211(5)	[45]	X	X	X
An9	Malvidin 3-acetylglucoside	37.2	535	331(100), 343(6)	**331:** 315(100), 299(99), 242(49), 270(38), 179(25), 139(9)	[45]	X	X	X
An10	Delphinidin 3-*p*-coumaroylglucoside	37.8	611	303(100)	**303:** 257(100), 275(5)	[45]	X	X	X
An11	Malvidin 3-6-caffeoyl-glucoside	37.9	655	331(100), 315(3), 242(3)	**331:** 315(100), 299(99), 270(75), 287(66), 179(38), 242(30)	[45]	X	X	X
An12	Cyanidin 3-*p*-coumaroylglucoside	38.4	595	287(100), 173(3), 185(3)	**287:** 213(100), 157(51), 231(51), 269(32), 185(20), 137(12)	[45]	X	X	X
An13	Petunidin 3- coumaroylglucoside	38.5	625	317(100), 302(4), 274(2)	**317:** 302(100), 274(18), 228(6)	[45]	X	X	X
An14	Peonidin 3-(6-*trans*-*p*-coumaroyl)-glucoside	40.2	609	301(100), 286(16), 201(3)	**301:** 286(100)	[45]	X	X	X
An15	Malvidin 3-*p*-coumaroylglucoside	40.7	639	331(100), 269(5), 315(4), 241(1)	**331:** 299(100), 315(98), 270(77), 242(37), 179(18), 253(6), 150(5)	[45]	X	X	X

* LOD, limit of detection; Rt, retention time.

## Data Availability

Not applicable.

## Sample Availability

Samples of the compounds are not available from the authors.
